# Formation of woody biomass is regulated by class III HD Zip genes

**DOI:** 10.1186/1753-6561-5-S7-P123

**Published:** 2011-09-13

**Authors:** Marcel Robischon

**Affiliations:** 1 Albert Ludwigs Universität Freiburg

## 

Secondary growth and the development of woody tissue is a key process in the formation of woody biomass. The gene family of Class III HDZip genes has been shown in the herbaceous Arabidopsis model to play a central role in regulating polarity and vascular development. While Arabidopis is a poor model for investigating processes of wood formation, in this project all poplar Class III HDZip genes were cloned and expressed in hybrid aspen as a tree model system. To circumvent an endogenous regulation mechanism involving microRNAs the sequences were also mutated to render them microRNA resistant. Lines expressing the mutated Poplar ortholog of the Arabidopsis Revoluta gene (Populus Revoluta PRE) show a spectacular phenotype with stunted growth, radialized and rolled leaves, and a double and at times triplication or quadruple layer of the xylem, suggesting the formation of multiple layers of cambium. ClassIII HDZip genes have thus been shown to be crucial for the formation of lignified tissue in trees.

